# Altered Balance of Reelin Proteolytic Fragments in the Cerebrospinal Fluid of Alzheimer’s Disease Patients

**DOI:** 10.3390/ijms23147522

**Published:** 2022-07-07

**Authors:** Inmaculada Lopez-Font, Matthew P. Lennol, Guillermo Iborra-Lazaro, Henrik Zetterberg, Kaj Blennow, Javier Sáez-Valero

**Affiliations:** 1 Instituto de Neurociencias de Alicante, Universidad Miguel Hernández-CSIC, 03550 Sant Joan d’Alacant, Spain; mlennol@umh.es (M.P.L.); guillermo.iborra@uclm.es (G.I.-L.); 2 Centro de Investigación Biomédica en Red sobre Enfermedades Neurodegenerativas (CIBERNED), 03550 Sant Joan d’Alacant, Spain; 3 Instituto de Investigación Sanitaria y Biomédica de Alicante (ISABIAL), 03010 Alicante, Spain; 4 Clinical Neurochemistry Laboratory, Sahlgrenska University Hospital, 413 45 Mölndal, Sweden; henrik.zetterberg@clinchem.gu.se (H.Z.); kaj.blennow@neuro.gu.se (K.B.); 5 Department of Psychiatry and Neurochemistry, Institute of Neuroscience and Physiology, The Sahlgrenska Academy at the University of Gothenburg, 413 90 Mölndal, Sweden; 6 Department of Neurodegenerative Disease, Institute of Neurology, University College London, London WC1E 6BT, UK; 7 UK Dementia Research Institute at UCL, London WC1E 6BT, UK; 8 Hong Kong Center for Neurodegenerative Diseases, Hong Kong, China

**Keywords:** reelin, proteolytic fragment, aggregate, cerebrospinal fluid, biomarker, Alzheimer’s disease

## Abstract

Reelin binds to the apolipoprotein E receptor apoER2 to activate an intracellular signaling cascade. The proteolytic cleavage of reelin follows receptor binding but can also occur independently of its binding to receptors. This study assesses whether reelin proteolytic fragments are differentially affected in the cerebrospinal fluid (CSF) of Alzheimer’s disease (AD) subjects. CSF reelin species were analyzed by Western blotting, employing antibodies against the N- and C-terminal domains. In AD patients, we found a decrease in the 420 kDa full-length reelin compared with controls. In these patients, we also found an increase in the N-terminal 310 kDa fragment resulting from the cleavage at the so-called C-t site, whereas the 180 kDa fragment originated from the N-t site remained unchanged. Regarding the C-terminal proteolytic fragments, the 100 kDa fragment resulting from the cleavage at the C-t site also displayed increased levels, whilst the one resulting from the N-t site, the 250 kDa fragment, decreased. We also detected the presence of an aberrant reelin species with a molecular mass of around 500 kDa present in AD samples (34 of 43 cases), while it was absent in the 14 control cases analyzed. These 500 kDa species were only immunoreactive to N-terminal antibodies. We validated the occurrence of these aberrant reelin species in an Aβ42-treated reelin-overexpressing cell model. When we compared the AD samples from *APOE* genotype subgroups, we only found minor differences in the levels of reelin fragments associated to the *APOE* genotype, but interestingly, the levels of fragments of apoER2 were lower in *APOE* ε4 carriers with regards to *APOE* ε3/ε3. The altered proportion of reelin/apoER2 fragments and the occurrence of reelin aberrant species suggest a complex regulation of the reelin signaling pathway, which results impaired in AD subjects.

## 1. Introduction

The ever-growing prevalence of Alzheimer’s disease (AD) is associated with the sporadic presentation of the disease [1,2]. Sporadic AD is a multifactorial disease with environmental contributing causes (mainly age); however, genetic risk factors are also important [3,4,5]. Amongst the numerous genes implicated in AD pathogenesis, the most prominent genetic risk factor is the apolipoprotein E (apoE) encoding gene, *APOE*. The apoE protein is secreted mainly by glial cells in the central nervous system [6], and it participates in cholesterol and lipid transport in the brain [7,8], amongst other physiological functions, including signaling through receptor interactions [6]. Three major apoE isoforms, apoE2, apoE3, and apoE4, are encoded by different alleles of human *APOE*, where the *APOE* ε3 allele (encoding apoE3) is the most common allele. The *APOE* ε2 allele (encoding apoE2) is the most protective against AD; however, it is also the least common allele. The *APOE*-ε4 allele (encoding apoE4), on the other hand, provides the highest risk of developing AD [9]. Interestingly, in humans, apoE isoforms form disulfide-linked homodimers that could be the native apoE form able to bind to receptors [10]. However, apoE4 is an exception, as it lacks a key cysteine residue. 

Reelin is a large, secreted glycoprotein composed of 3461 amino acids [11] that competes with apoE for receptor binding [12,13]. Reelin and apoE bind to the LDL receptor (LDLR) family, particularly apoE receptor 2 (apoER2), and the very low-density lipoprotein receptor (VLDLR). ApoER2 is the main reelin receptor into the brain. Thus, reelin/apoE-mediated signaling transduction occurs after binding to its receptors [14,15]. By binding to these receptors, reelin/apoE plays a key role in synaptic plasticity of the adult brain, primarily by mediating reelin signaling [16].

Studies from our group have shown that reelin protein and mRNA levels are elevated in AD subjects [17,18,19]. However, we have also demonstrated impaired reelin signaling in AD due to Aβ-mediated interference [19,20]. Nonetheless, the influence of the *APOE* genotype on reelin levels and function are not yet fully understood.

Full-length reelin forms homodimers, which are the species that drive efficient signal transduction [12,21,22,23]. We reported that Aβ may disrupt reelin from binding to receptors by hindering its capacity to form homodimers, thereby compromising the signaling process [20]. After interaction with apoER2, both reelin and apoER2 undergo proteolytic cleavage by metalloproteinases and secretases, respectively [24,25,26,27]. Nonetheless, reelin can undergo proteolytic processing through the activity of extracellular matrix metalloproteinases independently of its interaction with receptors [28]. Interestingly, truncated forms of reelin can form larger complexes that bind to reelin receptors, but they do not induce the signaling cascade activation efficiently [21], nor does the reelin monomer [29]. ApoER2 fragments resulting from proteolysis during receptor activation can also inhibit signaling [30]. Therefore, reelin and apoER2 proteolytic fragments may finetune the signaling pathway.

We postulate that the quantification of reelin fragments in the cerebrospinal fluid (CSF) can give a credible read-out of altered proteolytic processing of reelin and signaling impairment in AD subjects. We also discover an aberrant reelin species present in most AD subjects, regardless of *APOE* genotype. We validate the occurrence of these aberrant reelin species in an Aβ42-treated reelin-overexpressing cell model. In the present study, we also examine the effect of *APOE* genotype on the soluble levels of apoER2 fragments from subjects suffering from AD. We find differences in the proportions of these fragments in AD CSF that are associated with *APOE* genotype.

## 2. Results

### 2.1. Characterization of Reelin Species in AD CSF

**Figure 1:**
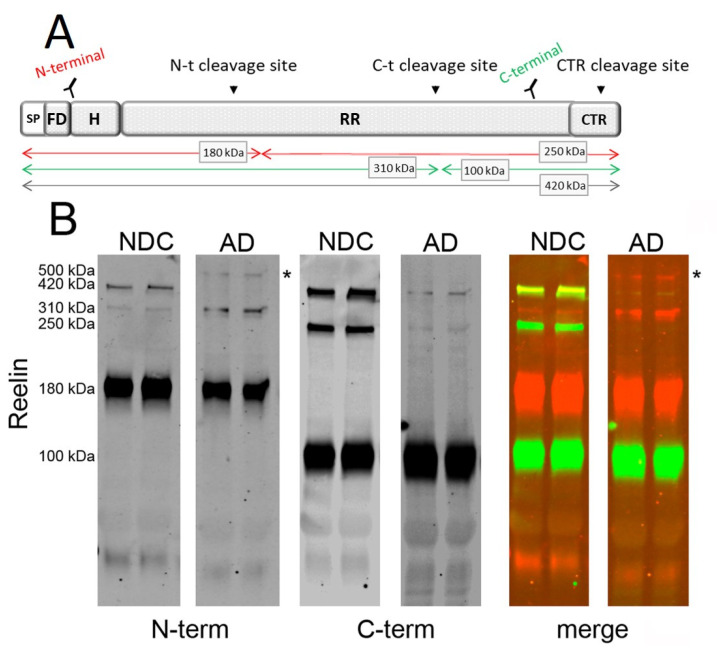
Reelin species present in human CSF. (**A**) Schematic representation of full-length reelin, the proteolytic cleavage site, and the epitope recognized by the antibodies used in the study. The reelin N-terminal region begins with a signal peptide (SP), an F-spondin-like domain (FD), and a hinge segment (H). This is followed by eight similar repeats (RR) separated by an EGF-like domain. The protein ends with a highly basic C-terminal region (CTR). Reelin cleavage at the N- and C-terminal regions leads to the formation of either 310 and 180 kDa N-terminal fragments of 100 kDa and 250 kDa C-terminal fragments, respectively. (**B**) The same human CSF samples from non-disease control (NDC) and AD subjects were simultaneously probed by Western blotting using multiplex fluorescence resolved with reelin N- and C-terminal antibodies. Representative blots of the N-terminal reelin bands (red) and C-terminal reelin bands (green) are shown, as well as simultaneous fluorescence (merge) demonstrating co-localization (yellow). * indicates ~500 kDa reelin immunoreactive band for N-terminal antibody present only in AD samples. The uncropped blot is included as Supplemental Figure S1.

Reelin undergoes cleavage by metalloproteinases at two major sites, called N-t and C-t sites (Figure 1A), resulting in the production of fragments whose relative abundance differs among tissues and fluids [31]. A third processing site within the C-terminal region was recently demonstrated (CTR cleavage; Figure 1A) [32].

Here, we analyzed CSF reelin species on 4–15% gradient SDS-PAGE with multiplex fluorescence using N- and C-terminal antibodies. In accordance with previous reports [17,18,24,33], a Western blot analysis of human CSF samples with an N-terminal antibody (mouse monoclonal) revealed full-length reelin (420 kDa), together with two proteolytic N-terminal fragments of 310 kDa (also known as NR6, a product of cleavage at the C-t site) and 180 kDa (also known as NR2, a product of cleavage at the N-t site) (Figure 1B). The C-terminal antibody (rabbit monoclonal) confirmed the identity of a 420 kDa full-length species, as well as fragments with the expected molecular mass [33], including a fragment of 100 kDa (also known as R7-8, a product of cleavage at the C-t site) and a less abundant 250 kDa fragment (also known as R3-8, a product of cleavage at the N-t site). Interestingly, while the N-terminal 180 kDa fragment was the most abundant, the correlative 250 kDa C-terminal fragment was the least abundant.

Intriguingly, we also detected the presence of an additional reelin immunoreactive band with a molecular mass around 500 kDa present exclusively in AD samples (Figure 1B). As far as we know, this 500 kDa reelin species has not been described previously. Moreover, this reelin species of 500 kDa was not immunoreactive to the C-terminal antibody, indicating that this species was not an alternative full-length isoform but, presumably, corresponded to SDS-stable complexes composed mainly of N-terminal fragments (Figure 1B). The higher sensitivity of the 420 kDa full-length reelin to proteolysis, including heating, limited the analysis of the complexes using alternative denaturing protocols since heating results in laddering [18,34].

### 2.2. Determination of Reelin Species in AD CSF

**Figure 2:**
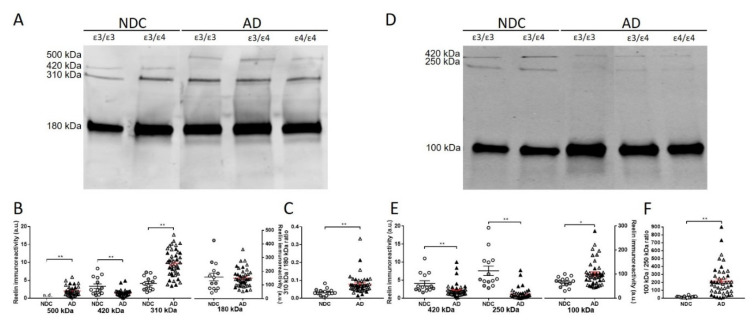
Characterization of reelin immunoreactive bands in CSF samples. (**A**) Representative blots of the reelin species detected in NDC and AD CSF samples using the N-terminal antibody. A species with a molecular mass of ~500 kDa was detected only in AD samples, together with the full-length species of 420 kDa and the 310 and 180 kDa proteolytic N-terminal fragments present in all the samples. (**B**) Densitometric quantification of the individual immunoreactivities of each reelin immunoreactive band detected with the N-terminal antibody. (**C**) Graph of the quotient obtained by dividing the level of immunoreactivity of the 310 kDa N-terminal fragment by the 180 kDa N-terminal fragment (310 kDa/180 kDa quotient). (**D**) Representative blot of reelin species immunoreactive to a C-terminal antibody in the CSF samples. (**E**) Densitometric quantification of reelin immunoreactivity from the 420 kDa full-length species and the 250 kDa and 100 kDa C-terminal fragments. (**F**) The ratio derived from the immunoreactivity for the 100 kDa C-terminal fragment with respect to the 250 kDa C-terminal fragment estimated in each sample (100 kDa/250 kDa quotient) is also shown. The samples are represented by the disease condition (NDC: circles; AD: triangles) and *APOE* genotype: *APOE* ε3/3 (white); *APOE* ε3/ε4 (grey); and *APOE* ε4/ε4 (black). There were 14 NDC subjects (9 *APOE* ε3 (7 *APOE* ε3/ε3 and 2 *APOE* ε3/ε2) and 5 *APOE* ε3/ε4) and 41 AD subjects (15 *APOE* ε3/ε3, 13 *APOE* ε3/ε4, and 15 *APOE* ε4/ε4). The data represent means ± SEM. * *p* < 0.05; ** p < 0.001; n.s. = nonsignificant; and n.d. = not detected. Graphs of comparisons between AD CSF samples subgrouped by *APOE* genotype are included as Supplemental Figures S2 and S3.

Next, we examined whether the levels of reelin fragments were altered in CSF samples from AD patients. Human CSF samples were from the Clinical Neurochemistry Laboratory (Mölndal, Sweden). All the AD patients fulfilled the NIAA-AA criteria for dementia [35] and were designated as AD according to CSF biomarker levels using cut-offs that were >90% specific for AD: Aβ42 < 550ng/L and total tau (T-tau) > 400ng/L [36] (see Table 1). When immunoblotting with the N-terminal antibody, we distinguished a full-length 420 kDa band and two N-terminal fragments of 310 and 180 kDa in all the CSF samples analyzed (Figure 2A). Interestingly, the 420 kDa full-length reelin was seen to be decreased (40%, *p* = 0.003) in AD samples compared with NDCs, whereas the 310 kDa fragment levels were increased (120%, *p* < 0.001). However, no significant differences were detected between the AD and NDC samples in the relative levels of the more abundant reelin species, the 180 kDa fragment (Figure 2B). The different tendency of the N-terminal fragments determined in AD CSF samples resulted in significant differences of a 310 kDa/180 kDa quotient (Figure 2C, *p* < 0.001).

Regarding the comparison between CSF samples subgrouped by *APOE* genotype, the changes in reelin species immunoreactive to the N-terminal antibody were maintained when comparing *APOE* ε3/ε3 AD cases (420 kDa decrease: 74%, *p* = 0.015; 310 kDa increase: 200%; *p* < 0.001) with NDC ε3/ε3 subjects. Given the difficulty finding age-matched *APOE* ε4/ε4 control subjects (low prevalence of this genotype in the general and healthy population), we could not compare AD *APOE* ε4/ε4 with control ε4/ε4 cases, and the analysis within the *APOE* ε3/ε4 genotype exhibited less statistical power because the size of the control group was small. Regardless, in the *APOE* ε3/ε4 AD subgroup, the full-length 420 kDa reelin tended to decrease (50%, *p* = 0.09), and the 310 kDa fragment increased significantly (104%; *p* = 0.019). When AD cases were compared between different *APOE* genotype subgroups, only the 310 kDa reelin displayed significant changes, being significantly lower (29%, *p* = 0.034) in subjects with the *APOE* ε4/ε4 genotype when compared to *APOE* ε3/ε3 subjects.

When immunoblotting with the C-terminal antibody (Figure 2D), the decreased levels of 420 kDa reelin were confirmed in the AD samples (Figure 2E; 51% decrease; *p* < 0.001). As expected, determination of the full-length reelin with the antibodies, both N- and C-terminal, displayed an effective correlation (r = 0.77, *p* < 0.001). The levels of the 100 kDa C-terminal fragment appeared increased in the AD samples (51%, *p* = 0.014) compared with NDC samples, whereas the 250 kDa fragment displayed a significant decrease (85%, *p* < 0.001). As a result, significant differences were also found for the 100 kDa/250 kDa quotient between the NDC and AD groups (Figure 2F; *p* < 0.001).

When the samples were subgrouped by *APOE* genotype, the changes in reelin species immunoreactive to the C-terminal antibody maintained their significance. Thus, in *APOE* ε3/ε3 subjects, the 420 kDa full-length reelin decreased in AD (51%, *p* < 0.001) compared to the NDC subgroup; the decrease was also significant when compared to *APOE* ε3/ε4 subjects (36%, *p* = 0.002). The increase in 100 kDa (58%, *p*= 0.043) and the decrease in 250 kDa (90%, *p*< 0.001) C-terminal fragments were still significant between the *APOE* ε3/ε3 AD and NDC subgroups. Similar changes were displayed in *APOE* ε3/ε4 subjects for the 250 kDa fragment (87% decrease in AD, *p* = 0.002), yet they failed to achieve significance for the 100 kDa fragment (68% increase in AD, *p* = 0.07). None of the C-terminal immunoreactive reelin species displayed significant changes between AD subjects subgrouped by *APOE* genotypes.

Furthermore, we confirmed the presence of the ~500 kDa reelin species (see Figure 1B) immunoreactive exclusively to the N-terminal antibody in AD cases in a total of 34 out of 43 CSF samples from AD patients across all the *APOE* genotypes, whilst the species was undetectable in the 14 NDC cases analyzed (Figure 2A).

Despite the fact that the AD subjects displayed differences in age compared with NDC subjects, the age of the subjects failed to correlate with the levels of the reelin species. Differences in reelin were not detected when the cases were subgrouped by gender.

There were no clear correlations between the levels of the reelin fragments with the levels of Aβ42 in either of the groups considered individually. Interestingly, in the NDC *APOE* ε3/ε3 subgroup, the levels of the major 180 kDa N-terminal fragment of reelin and T-tau correlated (*R* = 0.73, *p* = 0.020). This correlation was also significant in all the AD subjects subgrouped by *APOE* genotypes: ε3/ε3 (*R* = 0.61, *p* = 0.016), ε3/ε4 (*R* = 0.85, *p* < 0.001), and ε3/ε4 (T-tau with 180-kDa reelin: *R* = 0.59, *p* = 0.025). These results are in good agreement with a previous study also indicating that 180-kDa reelin levels correlated positively with T-tau protein in CSF [18].

### 2.3. Occurrence of the 500 kDa Reelin Species in Culture Media of Aβ42-Treated Cells

**Figure 3:**
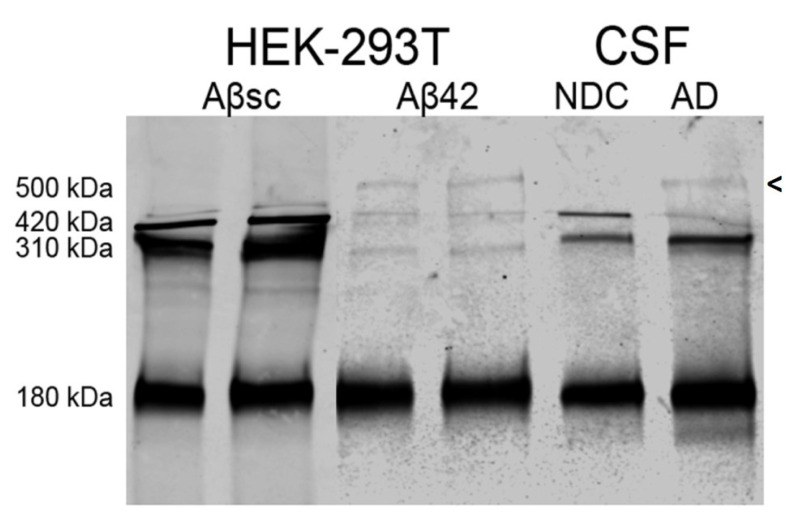
The 500 kDa reelin species was present in cell medium of HEK-293T treated with Aβ42. A representative blot showing the reelin immunoreactive bands detected with an N-terminal antibody from HEK-293T cells overexpressing reelin treated with Aβ42 or an Aβsc peptide. CSF samples from NDC and AD subjects were included to monitor the potential occurrence of the ~500 kDa species. When the cells were treated with Aβ42 peptide, a reelin immunoreactive band resembling the ~500 kDa band present in AD CSF was detected; this band was not present when the cells were treated with the Aβsc peptide. Arrowhead indicates the ~500 kDa band.

To validate the ~500 kDa reelin species and its association with the pathologic condition, we tested the potential occurrence of this species in a cellular model treated for 2 days with 2.5 µM Aβ42 or an Aβsc peptide, as described above. Culture media from HEK-293T cells over-expressing reelin were analyzed by Western blotting with an N-terminal reelin antibody. The characteristic N-terminal fragments were present in the culture media of cells over-expressing reelin, despite an important percentage of fragments being trapped with Aβ aggregates in pellets from the media of the cells, as described previously [19]. Interestingly, an immunoreactive reelin band of similar molecular mass to that of the ~500 kDa species identified in AD CSF samples appeared in cells treated with the amyloidogenic Aβ42 peptide, while the soluble reelin species present in the cells treated with the Aβsc peptide lacked the 500 kDa form (Figure 3).

### 2.4. Determination of CSF apoER2 in AD Subjects Subgrouped by APOE Genotype

**Figure 4:**
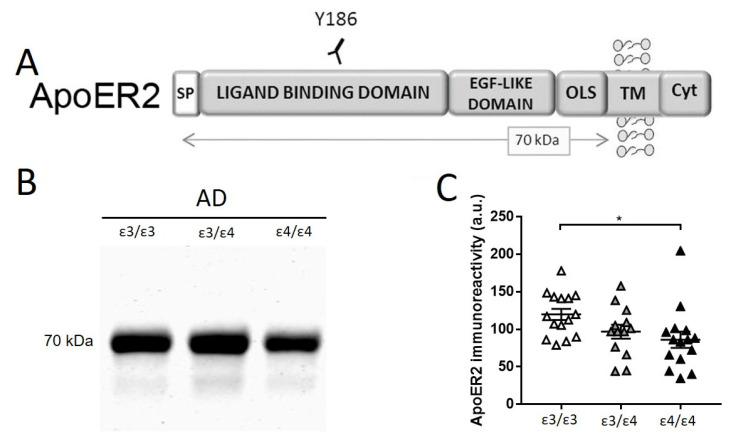
Characterization of apoER2 in CSF samples from AD. (**A**) Schematic representation of the apoER2 receptor and the epitope recognized by the Y186 antibody used in the study. ApoER2 is composed of a signal peptide (SP), followed by a ligand-binding domain containing an EGF-like region, an O-linked sugar domain (OLS), a transmembrane (TM) segment, and a cytoplasmic domain (Cyt). ApoER2 is processed by α-secretase upon ligand binding, generating a soluble ectodomain fragment (~70 kDa). (**B**) Representative blot of human CSF samples from AD subjects subgrouped by *APOE* genotype (15 *APOE* ε3/ε3, 13 *APOE* ε3/ε4, and 15 *APOE* ε4/ε4 subjects) and resolved with the indicated Y186 antibody. (**C**) Densitometric quantification and statistical analysis of the immunoreactivity and the 70 kDa ecto-apoER2 fragment. Samples are separated by *APOE* genotype: *APOE* ε3/3 (white), *APOE* ε3/ε4 (grey), and *APOE* ε4/ε4 (black). * *p* < 0.05.

The full-length apoER2 receptor has not been described in CSF; however, soluble apoER2 fragments were seen in human and ovine CSF [19]. This soluble fragment is generated after reelin binds to a receptor and induces apoER2 proteolytic cleavage [30]. The anti-apoER2 Y186 antibody specifically recognizes the entire ligand-binding ectodomain of the apoER2 receptor [37] (Figure 4A).

This antibody confirmed the existence of a ~70 kDa apoER2-soluble fragment, ecto-apoER2, in all the CSF samples analyzed (Figure 4B). Due to limitations in the available CSF sample volumes, the study of ecto-apoER2 levels was restricted to the AD collections subgrouped by *APOE* genotype. A previous report from our group demonstrated lower levels of ecto-apoER2 in AD CSF compared to those in age-matched controls [38], but this study did not address the influence of *APOE* genotype.

Significantly higher levels of ecto-apoER2 were detected in AD *APOE* ε3/ε3 subjects compared to *APOE* ε4/ε4 samples (28% decrease, *p* = 0.019), whereas in the comparison with *APOE* ε3/ε4 subjects, the trend was maintained but significance was not achieved (19% decrease, *p*= 0.073) (Figure 4C).

## 3. Discussion

In this study, we analyzed different reelin species in the CSF of patients suffering from AD. The 420 kDa reelin band, attributable to the full-length species, was seen to be decreased in the CSF from AD patients, while the levels of reelin fragments displayed distinct changes. Intriguingly, a 500 kDa species not yet described in other studies was seen to be present exclusively in AD samples.

Previous studies from our group have demonstrated increases in reelin protein and mRNA levels in brain frontal cortex extracts from AD patients and individuals with dementia associated with Down syndrome [17,18,19,39,40]. Reelin levels have also appeared to be increased in AD Tg2576 mutant mice and in cell cultures treated with amyloidogenic Aβ42 peptide [19,39]. Overall, our earlier studies have indicated that the more abundant 180 kDa reelin fragment appears to be slightly increased in the CSF of AD subjects, but this trend has not reached statistical significance in some reports [17,18,39]. In cells exposed to Aβ42 with increased cellular reelin protein levels, a reduced amount of soluble reelin was detectable in the culture media, probably because notable amounts of secreted reelin were “trapped” with the Aβ fibers [19]. Indeed, in the AD brain, considerable amounts of reelin appeared to be trapped in insoluble Aβ aggregates [40], and reelin immunostaining was observed in the amyloid plaques of APP/PS1 transgenic mice [41]. Thus, changes observed in the frontal cortex might not be reflected as clearly in the CSF from AD patients. It is worth mentioning that some studies have found contradictory results in which decreased reelin expression was associated with AD [42,43].

Several studies have indicated that dampened reelin signaling activity could contribute to the progression of AD (reviewed in [44]). Nonetheless, while reelin abundance could be elevated in the AD brain, we demonstrated that the interaction of reelin with Aβ hindered its biological activity [19]. A common feature in our previous studies has been the description of altered reelin glycosylation and oligomerization in the brain and CSF of AD subjects, which compromise the ability to transduce signals through the apoER2 receptor [18,20,39]. Thus, despite reelin levels being higher, the protein is inefficient in signal activation. The possibility that the generation of reelin and apoER2 proteolytic fragments after effective ligand-receptor interaction could also be affected has not been addressed until now.

In this context, the hypothesis of this study was that the analysis of reelin fragments in CSF could be informative of altered proteolytic processing and a subsequent impairment in signaling.

In previous studies addressing potential changes in the levels of reelin in AD CSF, the results have focused on the 180 kDa N-terminal fragment and the 100 kDa C-terminal fragment [17,18,33]. The full-length 420 kDa reelin and the less abundant 310 kDa fragment have been studied to a lesser extent due to difficulties in their detection due to weak staining. In this study, reelin species were detected with fluorescent-based imaging after SDS-PAGE and Western blotting. This technique provides a wider linear dynamic range than chemiluminescent detection [45], including a greater upper linear range of detection [46]. Moreover, all the analyses were performed on individual aliquots stored frozen at −80 °C, avoiding thawing–freezing cycles and limiting heating in the preparation of the samples for electrophoresis to 3 min since these pre-analytical and analytical factors can influence the measurement of reelin, particularly for full-length protein and large fragments [18,34]. Furthermore, in this study we optimized the resolution of the less abundant immunoreactive reelin bands of higher molecular mass by resolving the electrophoresis on 4-15% polyacrylamide-gradient gels (in previous analyses, 6% polyacrylamide gels have been used). These changes allowed us to enhance the resolution for a more reliable quantification of 420 kDa species and less abundant reelin fragments. We determined that the 420 kDa full-length reelin exhibited a pronounced reduction in the CSF of AD patients, whilst the 310 kDa N-terminal fragment presented a striking increase, suggesting a boosted rate of reelin proteolysis; however, the major N-terminal fragment of 180 kDa did not appear to be altered. Moreover, while the 100 kDa C-terminal fragment also increased, the 250 kDa fragment appeared clearly reduced in CSF samples from AD patients. Given that multiple proteolytic events allow the release of reelin fragments, the interpretation of these results is complex.

Experimental evidence has demonstrated that reelin is internalized following receptor binding [12] and subsequently suffers proteolytic processing [26], but little is known about the identity of the protease(s) in charge of the cleavage or the sequence of proteolytic events at N-t and C-t sites. Moreover, increasing evidence has indicated that extracellular matrix metalloproteinases act as reelin proteases. The association between dysregulated reelin proteolysis and disease progression is recurrent.

A study proposed that extracellular matrix ADAMTS-3 metalloproteinases were more than likely the proteases that cleaved reelin in vivo at the N-t site, originating the 180 kDa (NR2) fragment [47]. On the other hand, other studies have found that ADAMTS-2, ADAMTS-4, and ADAMTS-5 are potential enzymes that could cleave reelin at the N-t site (discussed in [28,48]). ADAMTS-4 and ADAMTS-5 are also able of cleaving reelin at the C-t site, whilst the serine protease tissue plasminogen activator (tPA), as well as meprin α and β, are other potential reelin cleavage proteases at its C-t site [49,50], originating the 310 kDa fragment that appeared to be particularly increased in this study. Interestingly, meprin β is also a sheddase for the amyloid precursor protein (APP) [51,52]; moreover, meprin β appeared to be increased in the brain of AD patients [53]. Meanwhile, the alternative C-t protease of reelin, tPA, appeared to be decreased or unchanged in the brain [54], CSF, and plasma of AD patients [55,56]. In this puzzling scenario, as mentioned above, it is assumed that the main proteolytic processing of reelin occurs following apoER2 binding and, subsequently, reelin fragments are re-secreted [26]. The major reelin fragment generated following reelin–apoER2 interaction is the 180 kDa (NR2) fragment. The identity of the protease(s) that cleaves reelin in the endosome remains unknown, and it may or may not be the same as the extracellular matrix metalloproteinases.

A previous in vitro experiment demonstrated that N-terminal fragments could form larger complexes that, despite binding well to receptors, did not induce efficient signaling [21]. Thus, differences in the relative abundance of 180 and 310 kDa N-terminal fragments in AD CSF could be related to a higher formation of these complexes in the pathological brain. Regardless, the large imbalance in the C-terminal fragments in AD CSF strongly suggests a dysregulation in reelin processing by extracellular matrix metalloproteinases.

Our improved determination of the largest reelin species enabled the identification of a novel ~500 kDa reelin immunoreactive species that has not yet been described. The ~500 kDa reelin species were immunoreactive to the N-terminal antibodies but were not recognized by the C-terminal antibody. We hypothesized that these reelin species probably represented part of the multimers of N-terminal fragments suggested by in vitro experiments [21]. These species are stable under denaturing conditions but are seen in very low amounts and are sensitive to heating during electrophoresis preparation, a common feature for all reelin species resulting in laddering [18,34], which complicates further analysis. These large reelin species were present in ~80% of the AD cases and in a HEK-293 cellular model over-expressing reelin treated with Aβ42 but were absent or very weak in the NDC cases and in the media of cells treated with a scrambled Aβ peptide. Despite the fact that HEK-293 is not a neuronal (-like) cell line, it is a widely used cellular model for over-expressing and studying reelin, as it shows the capacity to form N-terminal fragments and large multimers. In a previous study, reelin multimers were induced by chemical crosslinking [21]. Here, we replicated the occurrence of the ~500 kDa reelin in AD CSF by treating the cells with amyloidogenic Aβ42. Interestingly, complexes of several CSF proteins, such as presenilins [57,58], cholinesterase [59], and apoE [60,61], formed under amyloidogenic conditions appear to be particularly stable. Reelin interacts with Aβ both in vitro [62] and in vivo [19], as it is recruited into amyloid fibrils; thus, we hypothesized that Aβ played a direct role in the formation of the 500 kDa reelin species detected in AD CSF. A full characterization of said species is necessary for a correct interpretation of their potential pathological significance. As mentioned, we cannot discard that the 500 kDa reelin species were mostly composed by the 180 kDa fragments, which may offer an alternative explanation for the imbalance in the generation of N-terminal reelin fragments.

Furthermore, the *APOE* genotypes of the analyzed samples were considered in our analysis. In the AD group, only the 310 kDa reelin fragments exhibited lower levels in *APOE* ε4/ε4 when compared with *APOE* ε3/ε3 individuals. Interestingly, the levels of the ecto-apoER2 fragments also appeared to be decreased in *APOE* ε4/ε4 when compared with *APOE* ε3/ε3. ApoE competes with reelin for binding to apoER2 and VLDLR, and apoER2 also participates in the internalization of apoE-containing lipoprotein particles to incorporate cholesterol and other lipids that are essential for normal neuronal function in the cell [63,64]. Interestingly, apoE3 and apoE4 consistently inhibited reelin from binding to VLDLR and apoER2; this study also indicated that apoE interfered with the ability of reelin to activate a Dab1-dependent signaling pathway [12]. A later study also saw that, unlike reelin, apoE failed to elevate apoER2 processing [65], whereas others have found that the Dab1-dependent pathway was activated by apoE [66] and that apoE and apoER2 co-localized in endosomes [67].

Canonically, only dimeric ligand-binding induces effective signaling and the subsequent clustering of apoER2 [68]. The amino acid substitution of Cys-112 by Arg in apoE4 leads to the inability to form disulfide-linked homodimers and may impact some of the biological roles of apoE, particularly on receptor-binding activity [69]. Thus, despite similar affinities of apoE3 and apoE4 isoforms to the receptor [12,66], monomeric apoE4 can interact with apoER2 but may fail to drive signaling and subsequent reelin and apoER2 proteolytic processing; this would explain why the *APOE* ε4/ε4 individuals displayed lower levels of apoER2 and 310 kDa reelin compared with *APOE* ε3/ε3 individuals. Thus, apoE4 could block apoER2, impeding the binding of reelin. In this regard, was reported that apoE4 caused prolonged retention of the receptor inside the cell and impaired the signaling cascade [70]. Interestingly, in a recent study, the levels of membrane-bound apoER2 C-terminal fragments appeared to be significantly lower in AD extracts from advanced Braak stages of *APOE* ε4 noncarriers, but not in carriers [40]. Thus, we hypothesized that, in *APOE* ε4 carriers, apoER2 proteolysis was hampered, resulting in a reduction in ecto-apoER2 fragment release.

In *APOE* ε4/ε4 subjects, the generation of proteolytic fragments of reelin only appeared to be significantly reduced for the 320 kDa N-terminal fragment compared with *APOE* ε3/ε3, but nonsignificant trends were observed for reduced 180 kDa and increased 420 kDa levels. Similarly, the 100 kDa C-terminal fragment appeared to be nonsignificantly reduced in *APOE* ε4/ε4 compared with *APOE* ε3/ε3. Altogether, in agreement with decreased ecto-apoER2 generation, the results indicated that, in *APOE* ε4/ε4 AD patients, the efficiency of the reelin/apoE signaling pathways had a basal compromise due to the inability of apoE4 to biologically interact with the receptor. However, the effect of different *APOE* allelic variants on reelin signaling was more difficult to decipher due to the possibility that *APOE* allelic variants may also affect the activity and levels of reelin-cleaving metalloproteinases. For instance, apoE4 displayed a weaker ability to inhibit the function of matrix metallopeptidase 9 (MMP-9) than apoE2 or apoE3, given that MMP-9 expression and activity was elevated in the cerebrovasculature of both human and animal AD brains from specimens with the *APOE* ε4 genotype [71]. A study showed that MMP-9 could induce reelin processing at both the N-t and C-t sites indirectly through the activation of ADAMTS-4 [51]; however, another study failed to demonstrate the effect of MMP-9 inhibitor II on reelin cleavage [72].

In conclusion, we demonstrated the existence of an imbalance in reelin fragments in the CSF of AD patients, and we discovered an aberrant reelin species associated specifically with the amyloidogenic condition. This aberrant 500 kDa reelin species probably represented stable complexes of N-terminal fragments. Moreover, in AD patients with an *APOE* ε4/ε4 genotype, the generation of reelin and apoER2 fragments appeared to be distinctly different. Our results confirmed that reelin levels were altered associated with AD, probably reflecting an impairment in signaling. Our results also suggested that these changes may result, at least in part, from the activity of reelin-cleaving metalloproteinases. Interestingly, increasing reelin activity has been proposed as a therapeutic option for AD to protect against Aβ [62,73,74], as well as for others neuropsychiatric disorders [75]. In fact, reelin supplementation can enhance cognitive ability and synaptic plasticity [76]. However, for an effective acute activation of the reelin pathway, in addition to designing an adequate supplementation strategy, it seems necessary to monitor its efficiency in a pathological context where Aβ can interfere in the signaling, and the upregulation of reelin-cleaving metalloproteinases can reduce its effect. Thus, the determination of altered CSF reelin and apoER2 fragments may also be applicable as biomarkers for disease progression and for the efficiency of therapeutic agents targeting reelin.

## 4. Materials and Methods

### 4.1. Human CSF Samples

Human CSF samples were provided by the Clinical Neurochemistry Laboratory (Mölndal, Sweden). CSF was obtained through lumbar puncture and collected in polypropylene tubes. It was gently mixed to avoid gradient effects, centrifuged at 2000× *g* for 10 min at 4 °C to remove cells and other insoluble materials, aliquoted, and stored at −80 °C until use. The cohort studied consisted of CSF samples from 43 AD patients subgrouped according to their *APOE* genotype: 15 *APOE* ε3/ε3 subjects (4 female/11 male; 79 ± 2 of age), 13 *APOE* ε3/ε4 subjects (10 female/3 male; 78 ± 1 of age), 15 AD *APOE* ε4/ε4 subjects (9 female/6 male; 74 ± 1 of age), and 14 non-disease control (NDC) subjects (9 *APOE* ε3 (2 *APOE* ε3/ε2 and 7 *APOE* ε3/ε3; 5 female/4 male; 69 ± 2 of age) and 5 *APOE* ε3/ε4 (2 female/3 male; 62 ± 5 of age)). No sample calculation was performed. The patients were designated as AD according to CSF biomarker levels using cut-offs that were >90% specific for AD: Aβ42 < 550ng/L and total tau (T-tau) > 400ng/L [36] (see Table 1). The samples were retrospectively selected to balance age, sex, and *APOE* status.

All the AD patients fulfilled the NIAA-AA criteria for dementia [35]. Exclusion criteria were refusing lumbar puncture or neuropsychological investigation and current alcohol or substance misuse. No blinding was performed in this study. This study was not pre-registered. The variants rs7412 and rs429358 (which define the ε2, ε3, and, ε4 alleles) in the *APOE* gene were genotyped by mini-sequencing, as previously described in detail [77].

### 4.2. Cell Culture and Aβ Treatment

The effects of Aβ42 on cellular reelin levels were tested in HEK-293T cells that were stably transfected with reelin (kindly provided by Drs. E. Soriano and L. Pujadas, Department of Cell Biology, University of Barcelona, Barcelona, Spain). No further authentication was performed in the laboratory. A maximum of 5 cell passages was used. To obtain conditioned cell culture medium, 2 × 10^6^ cells/dish were grown in six-well plates in Dulbecco’s modified Eagle’s medium (DMEM) supplemented with 10% fetal bovine serum (FBS; Gibco), 100 μg/mL penicillin/streptomycin (Gibco), and 250 µg/mL G418 (Sigma). After 24 h, the medium was changed to a modified Eagle’s Minimum Essential Media (Opti-MEM; Gibco), and the cells were treated with 2.5 µM β-amyloid 1-42 peptide (Aβ42) or a β-amyloid scrambled control peptide (Aβsc; AIAEGDSHVLKEGAYMEIFDVQGHVFGGKIFRVVDLGSHNVA) (American Peptide Co., USA) for 2 consecutive days without changing the medium. After 3 days, the cell medium was filtered through 0.2 μm pores and concentrated with an Amicon Ultra 100 kDa size exclusion filter (Merk Millipore, Darmstadt, Germany), followed by analysis using Western blot [19,20].

### 4.3. Western Blot

For the analysis of reelin under denaturing conditions, CSF samples (13 μL) or culture media (13 μL) were resolved on 4–15% gradient SDS-PAGE (Mini-PROTEAN^®^ TGX™ Precast Gels; Bio-Rad) and transferred to 0.45 µm nitrocellulose membranes (Bio-Rad). The samples were boiled at 98 °C in reducing Laemmli SDS sample buffer containing 2-mercaptoethanol (Thermo Scientific^TM^) for only 3 min, given that storage and heat affect reelin determination noticeably, particularly through fragmentation of the full-length species [18,34]. Likewise, freezing–thawing cycles before electrophoresis were avoided. The transferred proteins were detected with fluorescent-based imaging using an anti N-terminal reelin mouse monoclonal antibody (1:1000; Millipore MAB5366; epitope 164–189; clone 142) or an anti C-terminal reelin rabbit monoclonal antibody (1:1000; Abcam ab139691; epitope 3250–3350). The transferred proteins were also detected using a Y186 antibody for apoER2 (rabbit monoclonal anti-N-terminal apoER2 186 antibody; 1:4000; generously provided by Prof. Johannes Nimpf, Department of Medical Biochemistry, Max F. Perutz Laboratories, Medical University of Vienna, Vienna, Austria). Pre-stained molecular weight markers for electrophoresis were obtained from Thermo Scientific (PageRuler™ Plus, ref code# 26,620, or HiMark pre-stained protein standard, ref code# LC5699). A control CSF sample that was previously aliquoted was included into every blot and was used to normalize the immunoreactive signal between immunoblots. The immunoreactivity of each individual band of reelin was corrected with the immunoreactivity of the 180-kDa band (in the case of N-terminal bands) or with the 100-kDa band (in the case of C-terminal bands) from the control sample, reducing interblot variability and allowing for comparisons across blots. For apoER2, the immunoreactivity of the 70 kDa band was also corrected using the same strategy. The blots were then probed with the appropriate conjugated secondary IRDye antibodies (IRDye 680RD goat anti-mouse and IRDye 800RD goat anti-rabbit) and recorded with an Odyssey CLx Infrared Imaging system (LI-COR Biosciences GmbH). Multiplex fluorescence with the two independent antibodies served to simultaneously assess N- and C-terminal reelin. Band intensities were analyzed using LI-COR software (Image Studio Lite). To estimate the quotients between the different reelin bands of each sample, the unprocessed immunoreactivity for each of the bands was considered. All the samples were analyzed in duplicate. All the samples were determined at least in duplicate (duplicates in separate gels) and distributed in the gels to ensure comparison by disease condition and *APOE* genotype. The distribution of the samples in the gels was performed by a member of the team, and the experiment was performed by another, the experimenter, in a blind way.

### 4.4. Measurement of T-tau and Aβ42 by ELISA

The levels of the AD core biomarkers T-tau and Aβ42 were measured in the CSF using INNOTEST ELISAs (Fujirebio Europe, Gent, Belgium). All the samples were analyzed as part of a clinical routine by board-certified laboratory technicians following strict procedures for the batch-bridging, analysis, and quality control of the individual ELISA plates [78].

### 4.5. Statistical Analyses

All the data were analyzed using GraphPad Prism 6.0 (GraphPad Software, San Diego, CA, USA). The Kolmogorov–Smirnov test was used to analyze the distribution of each variable. ANOVA was used for parametric variables, and the Kruskal–Wallis test was used for nonparametric variables for comparison between the groups. Student’s *t*-test for parametric variables and the Mann–Whitney U test for nonparametric variables were employed for comparison between the means of two groups and for determining *p*-values. Outliers were not excluded. For correlations, Pearson and Spearman tests were used. The results are presented as means ± SEM.

## Figures and Tables

**Table 1:** Clinical and demographic data, as well as classic CSF biomarkers, for the samples used in this study. F, female; M, male. The data represent means ± SEM. Significant difference was * *p* < 0.0001, with respect to the NDC group.

CSF Cohort							
	Control			Alzheimer’s Disease			
*APOE*	ε3/3	ε3/4	All	ε3/3	ε3/4	ε4/4	All
N	9	5	14	15	13	15	43
Age (Years)	69 ± 2	62 ± 5	67 ± 3	79 ± 2	78 ± 1	73 ± 1	77 ± 1 *
Age (Range)	60–81	44–75	44–81	62–88	69–84	63–83	62–88
Female/Male	5/4	2/3	7/7	11/4	10/3	9/6	31/14
CSF Aβ42 (pg/mL)	845 ± 96	746 ± 121	804 ± 74	470 ± 13 *	484 ± 9 *	419 ± 21	457 ± 10 *
CSF Total Tau (pg/mL)	317 ± 53	303 ± 34	312 ± 35	816 ± 88 *	1004 ± 127 *	731 ± 53	840 ± 52 *

## Supplementary Materials

The following supporting information can be downloaded at: https://www.mdpi.com/article/10.3390/ijms23147522/s1.

## Abbreviations

Aβ: β-amyloid protein; AD: Alzheimer’s disease; apoE: apolipoprotein E; apoER2: apolipoprotein E receptor 2; CSF: cerebrospinal fluid; NDC: non-disease control; T-tau: total tau protein.
