# Supplementary material for: Altered Balance of Reelin Proteolytic Fragments in the Cerebrospinal Fluid of Alzheimer’s Disease Patients
Source: Int J Mol Sci. 2022 Jul 7;23(14):7522. doi: 10.3390/ijms23147522 (PMC9318932; doi:10.3390/ijms23147522)
Supplement: Supplementary file 1 [file ijms-23-07522-s001.zip › ijms-1764412-supplementary.pdf]

Article

Altered Balance of Reelin Proteolytic Fragments in the Cerebrospinal Fluid of Alzheimer's Disease Patients

Inmaculada Lopez-Font, Matthew P. Lennol, Guillermo Iborra-Lazaro, Henrik Zetterberg, Kaj Blennow and Javier Sáez-Valero

Supplemental information

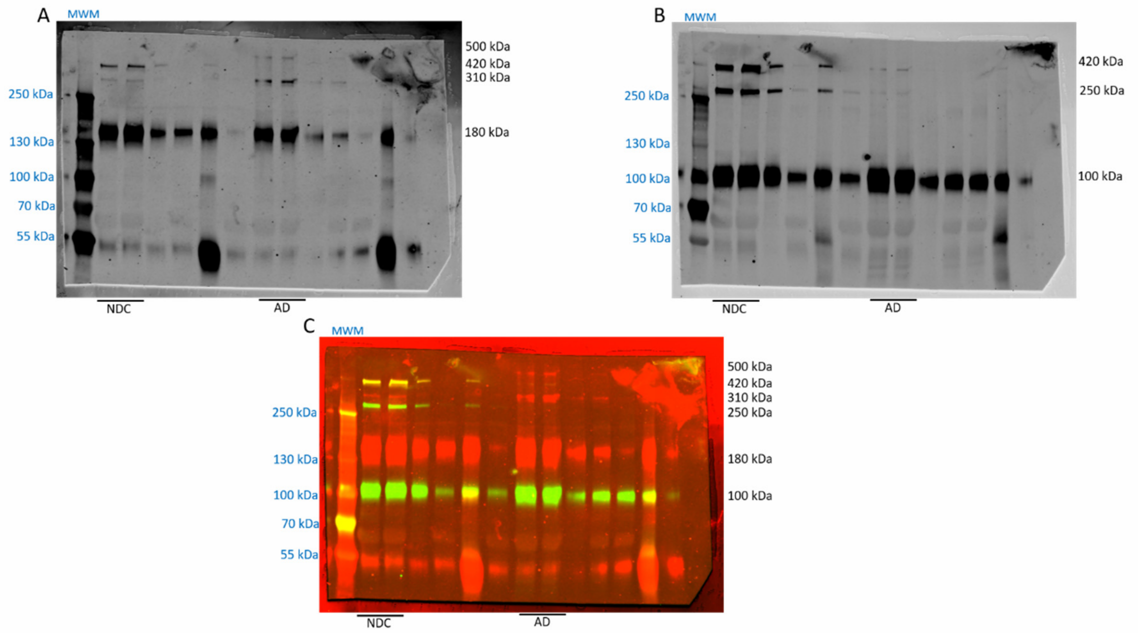

Supplementary Figure S1. Full image of representative blot selected for Figure 1. Please note that lanes 2 and 3 (NDC) and 8 and 9 (AD) were used. (**A**) N-terminal; (**B**) C-terminal; (**C**) Merge of N-terminal (red) and C-terminal (green) anti-reelin antibodies. See Figure 1 for further details. In this blot we used PageRuler™ Plus as pre-stained molecular weight markers (MWM) for electrophoresis (the molecular mass of the markers is indicated in blue), in other blots the HiMark pre-stained protein standard served to estimate more accurately the approximate molecular mass of the 420 and 500-kDa reelin species.

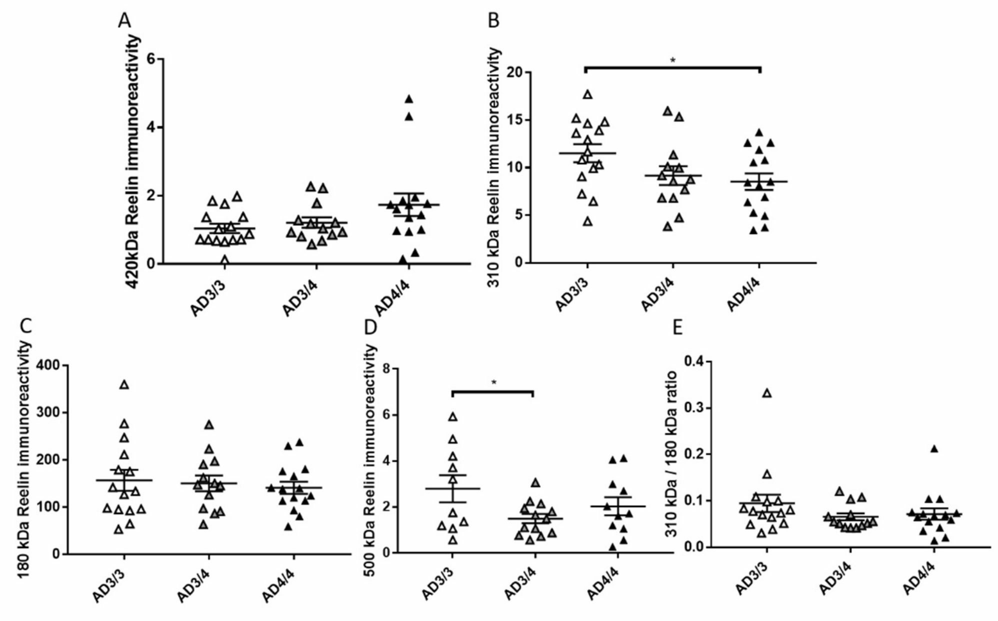

Supplementary Figure S2. Graphs from N-terminal reelin species for AD samples, separated by *APOE* genotype. (**A**) Levels of 420 kDa species; (**B**) 310 kDa species; (**C**) 180 kDa species. (**D**) 500 kDa species. (**E**) The ratio for 310kDa/180kDa species. White triangles: ADε3/ε3; Grey triangles: ADε3/ε4; Black triangles: ADε4/ε4. See Figure 2 for further details. *: $p<0.05$

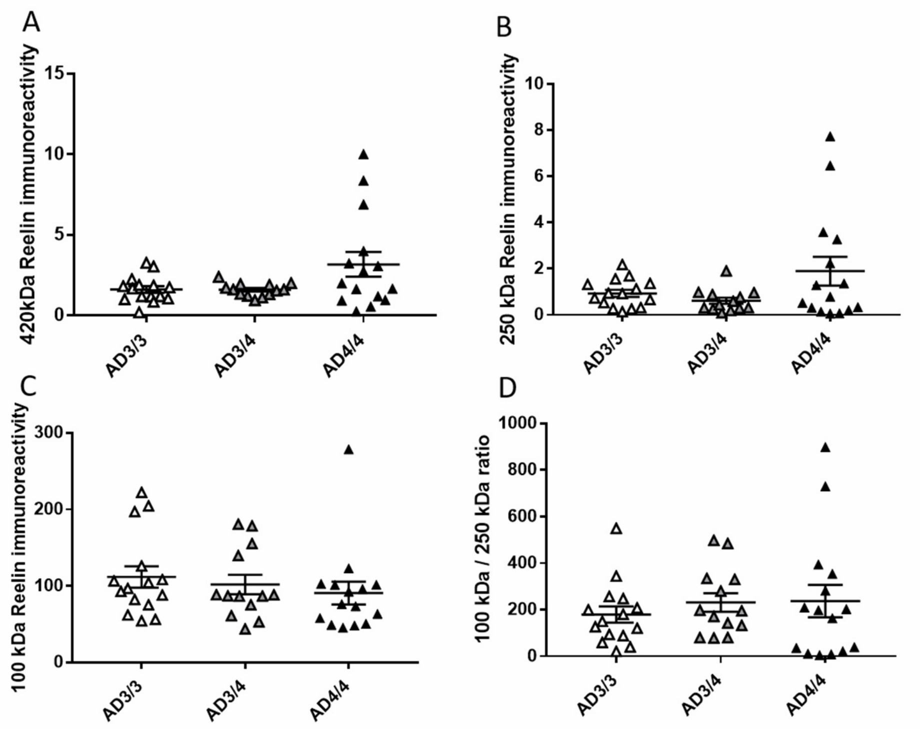

Supplementary Figure S3. Graphs from N-terminal reelin species for AD samples, separated by *APOE* genotype. (**A**) Levels of 420 kDa species; (**B**) 250 kDa species; (**C**) 100 kDa species. (**D**) The ratio for 100kDa/2500kDa species. White triangles: ADε3/ε3; Grey triangles: ADε3/ε4; Black triangles: ADε4/ε4. See Figure 2 for further details.
